# Pulmonary metastases from malignant melanoma showing ground-glass opacity nodules

**DOI:** 10.1007/s11604-025-01745-1

**Published:** 2025-02-05

**Authors:** Keisuke Todoroki, Satoshi Kawakami, Yukiko Kiniwa, Shiho Asaka, Hideki Endoh, Yasunari Fujinaga

**Affiliations:** 1 Department of Radiology, Shinshu University School of Medicine, 3-1-1 Asahi, Matsumoto, 390-8621 Japan; 2 Department of Dermatology, Shinshu University School of Medicine, 3-1-1 Asahi, Matsumoto, 390-8621 Japan; 3 Department of Laboratory Medicine, Shinshu University School of Medicine, 3-1-1 Asahi, Matsumoto, 390-8621 Japan; 4 Department of Laboratory Medicine and Pathology, Life Science Research Center, Nagano Children’s Hospital, 3100 Toyoshina, Azumino, 399-8205 Japan; 5 Department of Thoracic Surgery, Saku Central Hospital Advanced Care Center, 3400-28 Nakagomi, Saku, 385-0051 Japan

**Keywords:** Lung metastasis, Malignant melanoma, Ground-glass opacity nodule, Computed tomography

## Abstract

**Purpose:**

To investigate the frequency and characteristics of pulmonary metastases from malignant melanoma presenting as ground-glass opacity nodules (GGNs) on chest computed tomography (CT).

**Material and methods:**

A total of 354 patients with malignant melanoma who underwent chest CT for staging or follow-up were selected. We reviewed the CT images and enrolled 87 patients with lung metastases. Two radiologists evaluated the nodularity of the lung metastases (solid nodules or GGNs). Additionally, the tumor doubling time and disease type (mucosal, cutaneous, or acral melanomas) were analyzed.

**Results:**

GGNs were observed in 13 of 87 (14.9%) patients. The tumor doubling time was 52.0 ± 33.5 days (range: 10.9–111 days) for GGNs and 43.8 ± 27.5 days (range: 9.4–115.3 days) for solid nodules. GGNs changed to solid nodules in 54.5% of patients with increased GGN metastasis. More patients in the GGN group (patients whose metastases included GGNs) had mucosal melanomas than acral melanomas (p = 0.0478); however, no significant difference was observed in the frequency of mucosal and cutaneous melanomas (p = 0.0670). Similarly, the proportion of patients in the GGN-dominant pattern group (patients with GGNs only or more GGNs than solid nodules) who had mucosal melanomas was more than that of patients with acral and cutaneous melanomas (mucosal melanoma vs. acral melanoma, p = 0.0342; mucosal melanoma vs. cutaneous melanoma, p = 0.0344).

**Conclusions:**

Lung metastases from malignant melanoma sometimes appear as GGNs on CT, with a frequency of 14.9% in this study. If lung metastasis is observed as a GGN, the tumor doubling time may be useful for differentiating lung metastasis of malignant melanoma from lung adenocarcinoma.

## Introduction

Pulmonary metastases typically appear as multiple, rounded, solid nodules but sometimes present with atypical findings. The air-space pattern (consolidation, ground-glass opacities, and halo sign) is an atypical metastasis reported in pancreatic, colon, small bowel, breast, and ovarian cancers [1–4]. Several cases of pulmonary metastases from malignant melanoma occurring as ground-glass nodules (GGNs) have been reported, and well-differentiated lung adenocarcinoma and lung metastases of malignant melanoma can both appear as single or multiple GGNs on computed tomography (CT) [5–9].

However, the treatment strategies for well-differentiated lung adenocarcinoma and lung metastases of malignant melanoma are completely different. Surgery is the standard treatment for early-stage lung adenocarcinoma [10], whereas systemic therapy is mainly used for malignant melanoma metastases to the lungs [11]. When GGNs are detected in patients with malignant melanoma, differentiating between them is important. However, no reports have investigated the CT findings or the frequency of pulmonary metastases as GGNs.

Therefore, this study aimed to evaluate the prevalence and characteristics of pulmonary metastases in patients with malignant melanoma presenting as GGNs.

## Materials and methods

This retrospective study was approved by the Institutional Review Board of Shinshu University School of Medicine (ethics approval number: 5783), which waived the requirement for informed consent. The study was performed per the ethical standards as laid down in the 2013 Declaration of Helsinki.

### Patient selection

This single-center retrospective study was conducted from January 1, 2012, to December 31, 2021 (a 10-year period). The inclusion criteria were as follows: (i) patients with malignant melanoma whose CT images were stored in our picture archiving and communication system and (ii) patients who were diagnosed with malignant melanoma based on pathological findings after surgical operations or biopsies. We included 173 men and 170 women with a mean ± standard deviation (SD) age of 66.5 ± 15.4 years (range: 19–95 years).

The exclusion criteria were as follows: (i) patients without lung metastases, (ii) absence of thin CT sections (≤ 3 mm), and (iii) patients with uveal melanoma.

**Fig. 1:**
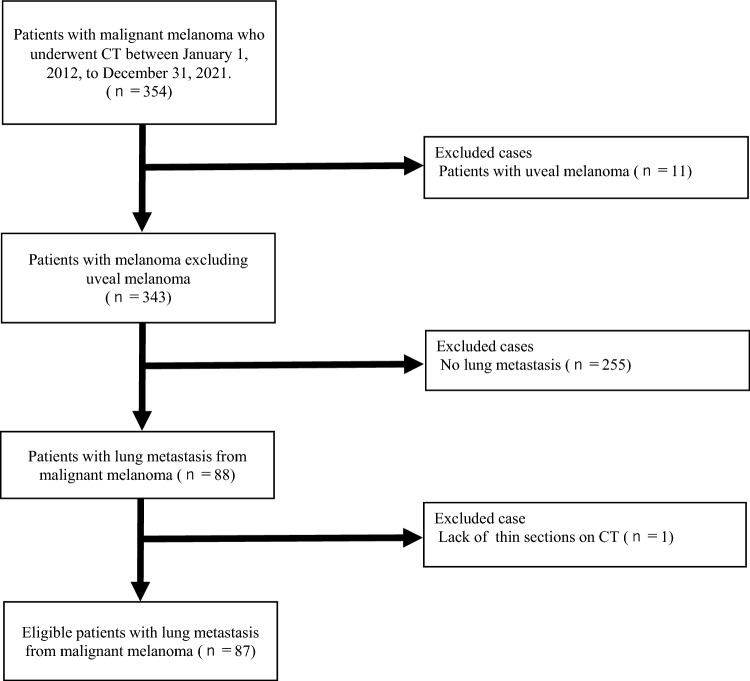
Flowchart of this study

Pulmonary metastasis was observed in 88 patients. However, one patient was excluded owing to the lack of available thin sections (≤ 3 mm) on CT. The final study population comprised 87 patients with lung metastases (Fig. 1, Table 1). The patients included 38 men and 49 women with a mean ± SD age of 66.1 ± 15.7 years (range: 19–88 years).

**Table 1:** Characteristics of patients with malignant melanoma

Patients with malignant melanoma	N = 343 (% is shown in parentheses)
Malignant melanoma type	
Mucosal	60 (17.5%)
Cutaneous	140 (40.8%)
Acral	130 (37.9%)
Unknown	13 (3.8%)

Patients with lung metastases	N = 87
Clinical prognostic stage at first diagnosis	
0	1 (1.1%)
IA	1 (1.1%)
IB	3 (3.4%)
IIA	4 (4.6%)
IIB	10 (11.5%)
IIC	8 (9.2%)
III	39 (44.8%)
IV	16 (18.4%)
cTXN0M0	4 (4.6%)
Unknown	1 (1.1%)
No. of GGN group/SN group in each malignant melanoma type	
Mucosal (n = 18)	6/12*
Cutaneous (n = 36)	4/32
Acral (n = 26)	2/24*
Unknown (n = 7)	1/6
No. of GGN-dominant pattern/SN-dominant pattern in each malignant melanoma type	
Mucosal (n = 18)	5/13†‡
Cutaneous (n = 36)	2/34†
Acral (n = 26)	1/25‡
Unknown (n = 7)	0/7

*GGN* ground-glass nodule, *SN* solid nodule

*p = 0.0478, †p = 0.0344, ‡p = 0.0342

*p = 0.0478 is mucosal vs acral,not include cutaneous. ‡p = 0.0342 is mucosal vs acral, not include cutaneous

Patients were staged according to the eighth edition of the American Joint Committee on Cancer [12]. As it was difficult to accurately determine the pathological stage in all cases (for example, if surgery had not been performed), a clinical staging system was employed in this study. Accurate staging was not possible in some patients who underwent only a biopsy or were transferred to our hospital.

### Diagnostic criteria for lung metastasis

Pulmonary metastasis was diagnosed when at least one of the following criteria was present: (i) CT showed typical imaging features such as multiple, rounded, solid nodules; (ii) multiple nodules that showed growth on serial follow-up CT or shrunk with systemic therapy; and (iii) histopathological diagnosis of pulmonary metastasis of malignant melanoma was obtained by surgery.

### CT protocol and image analysis

CT examinations were performed at our hospital using the 256-detector row CT scanner (Revolution CT) and 64-detector row CT scanner (Revolution HD, Light speed VCT, Discovery CT750 HD; GE HealthCare, Milwaukee, WI, USA) with a slice thickness of 0.625–2.5 mm. Nine patients with lung metastases had follow-up CT scans taken at other hospitals, of whom two patients were scanned using a 320-detector row CT scanner (Aquilion ONE, Canon Medical Systems, Otawara, Japan; slice thickness: 1.0 mm), one patient using a 128-detector row CT (SOMATOM Definition Flash, Siemens Healthineers, Forchheim, Germany; slice thickness: 2.0 mm), two patients using a 64-detector row CT (Discovery CT750 HD, GE HealthCare; slice thickness: 1.25–2.5 mm), one patient using a 64-detector row CT (Aquilion, Canon Medical Systems; slice thickness: 2.0 mm), two patients using a 32-detector row CT (LightSpeed Pro 32, GE HealthCare; slice thickness: 2.5 mm), and one patient using a 16-detector row CT (Aquilion, Canon Medical Systems; slice thickness: 2.0 mm). The window setting for all CT scans was −550–1500 Hounsfield units. Contrast studies were not included in the chest CT evaluations for nodules.

The CT images were assessed by two chest radiologists (K.T. and S.K. with 5 and 28 years of experience in chest radiology imaging, respectively) in consensus.

Pulmonary metastases were classified into four types: type 1, only solid nodules; type 2, solid nodules and GGNs, where the number of solid nodules was more than the GGNs; type 3, solid nodules and GGNs, where the number of GGNs was equal or more than the solid nodules; and type 4, only GGNs.

**Fig. 2:**
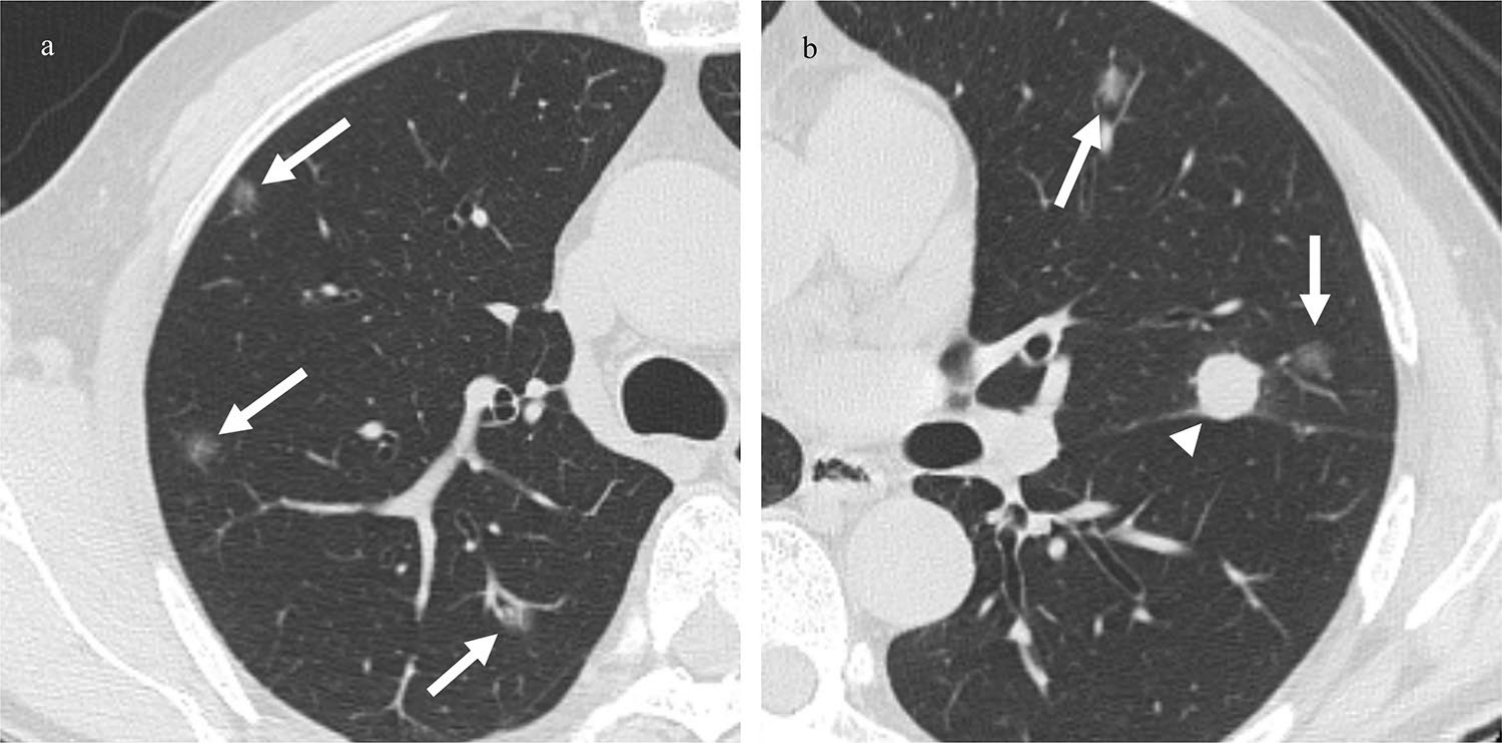
A 56-year-old man with mucosal melanoma with a GGN-dominant pattern. **a**, **b**. Follow-up CT 10 months after the operation shows multiple metastases as GGNs in bilateral lungs (white arrows). **b**. Some metastases appeared as solid nodules (white arrowhead). GGN, ground-glass nodule; CT, computed tomography

**Fig. 3:**
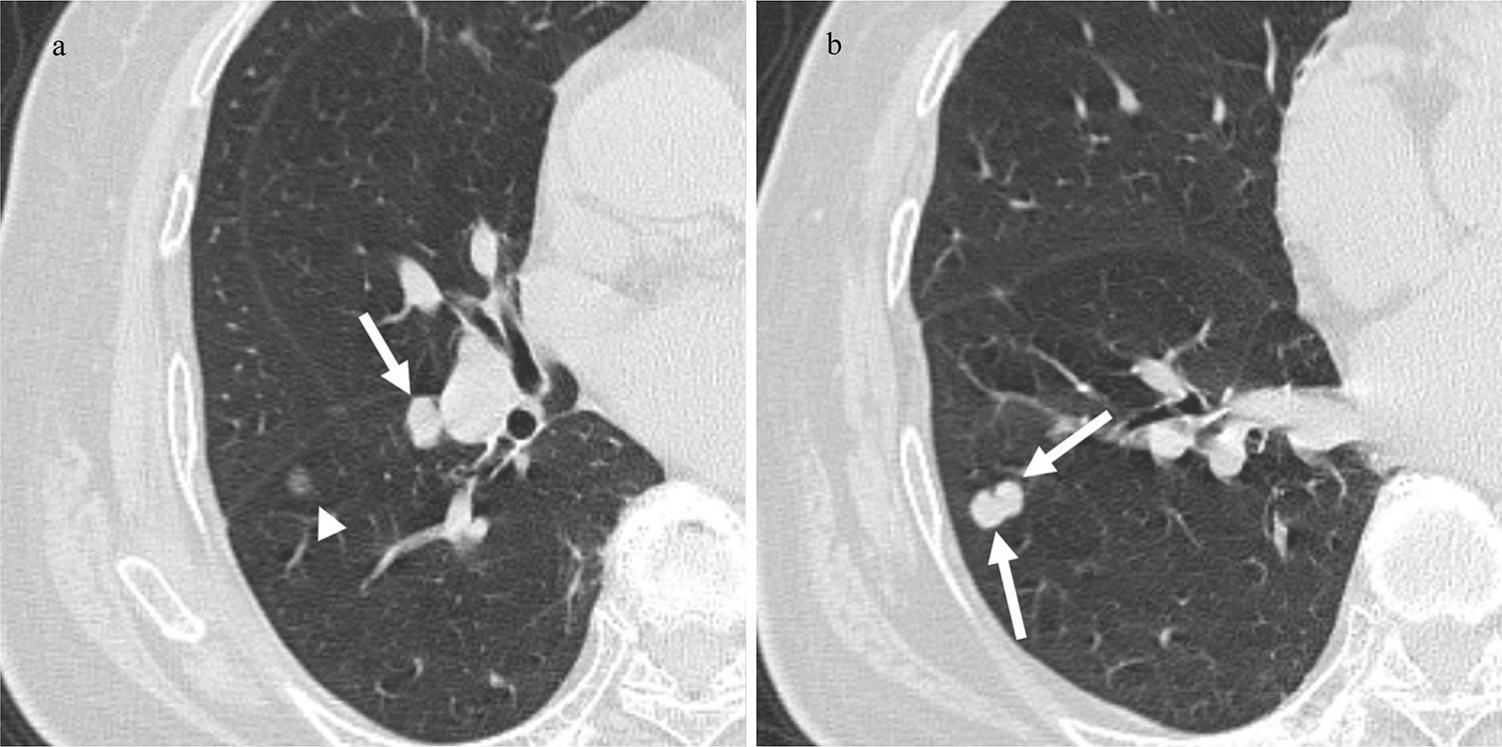
An 84-year-old woman with acral melanoma with a solid nodule-dominant pattern. **a**, **b**. Follow-up CT 9 months after the operation shows multiple metastases as solid nodules in bilateral lungs (white arrows). a. Some metastases appeared as GGNs (white arrowheads). After 5 months, some of the GGNs became solid nodules (not shown)

Based on the presence of GGNs, patients with lung metastases were classified into two groups: patients whose metastases included GGNs (GGN group; types 2, 3, and 4) and those whose metastases included only solid nodules (SN group; type 1). Additionally, focusing on the dominance of solid nodules or GGNs, we classified the GGN group into two subtypes: patients with GGNs only or more GGNs than solid nodules (GGN-dominant pattern; types 3 and 4) (Fig. 2) and patients with only solid nodules or more solid nodules than GGNs (SN-dominant pattern; types 1 and 2) (Fig. 3).

Nodule characterization and tumor doubling time were based on the initial CT that showed lung metastasis. We determined the size of GGN as the total length including the ground-glass component. The size of the solid nodule was defined as its diameter. In the GGN group, the largest GGN was selected regardless of the size of the solid nodule. In the SN group, the largest solid nodule was selected. Tumor doubling time was calculated in cases in which no systemic therapy was administered between the two CT scans where the tumor size was measured (GGN group, six patients; SN group, 32 patients). Tumor doubling times were calculated using the method originally described by Schwartz [13].

$$DT = t \times \,\log^2 / \;3 \; \times \,\log \left( {{\raise0.7ex\hbox{${Dt}$} \!\mathord{\left/ {\vphantom {{Dt} {D0}}}\right.\kern-0pt} \!\lower0.7ex\hbox{${D0}$}}} \right)$$

where DT = tumor doubling time; t = the time of the second measurement of the tumor size.

D0 = the initial diameter of the tumor; Dt = the tumor diameter at time t.

### Classification of malignant melanoma

The classification of malignant melanoma is based on ultraviolet radiation, the cell of origin, and characteristic recurrent genomic alterations [14]. The analysis was conducted by classifying into mucosal, cutaneous, and acral melanomas and unknown according to the WHO 2018 classification of cutaneous melanocytic neoplasms: suggestions from routine practice [15]. Uveal melanoma differs from cutaneous melanoma in terms of genomic abnormalities, metastatic mode, and lack of effective drugs and was, therefore, excluded from this study. Several other rare types of malignant melanomas were also not included in this study. The study included 60 patients with mucosal melanoma, 140 with cutaneous melanoma, 130 with acral melanoma, and 13 unknown (Table 1).

### Statistical analysis

All statistical analyses were performed using EZR [16], a graphical user interface for R. It is a modified version of the R commander designed to add statistical functions frequently used in biostatistics.

To determine the relationship between melanoma type and GGN, Fisher’s exact test was performed to compare the GGN group with the SN group and the GGN-dominant pattern with the SN-dominant pattern. Statistical significance was set at p < 0.05.

## Results

In this study, 74 patients were classified as type 1, five were classified as type 2, four as type 3, and four as type 4 (Table 2). In the type 4 group, all GGNs were pure GGNs at the initial CT that showed lung metastasis. The frequency of GGN, patterns of lung metastases, and pathological types of malignant melanomas are shown in Tables 1 and 2. The median time from the date of initial diagnosis of malignant melanoma by surgery or biopsy to the date of appearance of lung metastasis was 383.5 days (range: 0–7342 days, excluding one person for whom the exact date of surgery was unknown). The mean tumor doubling time was 52.0 ± 33.5 days (range: 10.9–111 days) in the GGN group and 43.8 ± 27.5 days (range: 9.4–115.3 days) in the SN group.

**Table 2:** Types, groups, and patterns of lung metastases from malignant melanoma

CT findings		Type; No. of patients (%)	Group; No. of patients (%)	Pattern; No. of patients (%)
Solid nodules only		1;74 (85.1%)	SN; 74 (85.1%)	Solid nodule dominant; 79 (90.8%)
Solid Nodule and GGNs	No. of solid nodules > GGNs	2;5 (5.7%)	GGN; 13 (14.9%)	
	No. of solid nodules ≤ GGNs	3;4 (4.6%)		GGN dominant;8 (9.2%)
GGNs only		4;4 (4.6%)		

GGN, ground-glass nodule; SN, solid nodule

**Fig. 4:**
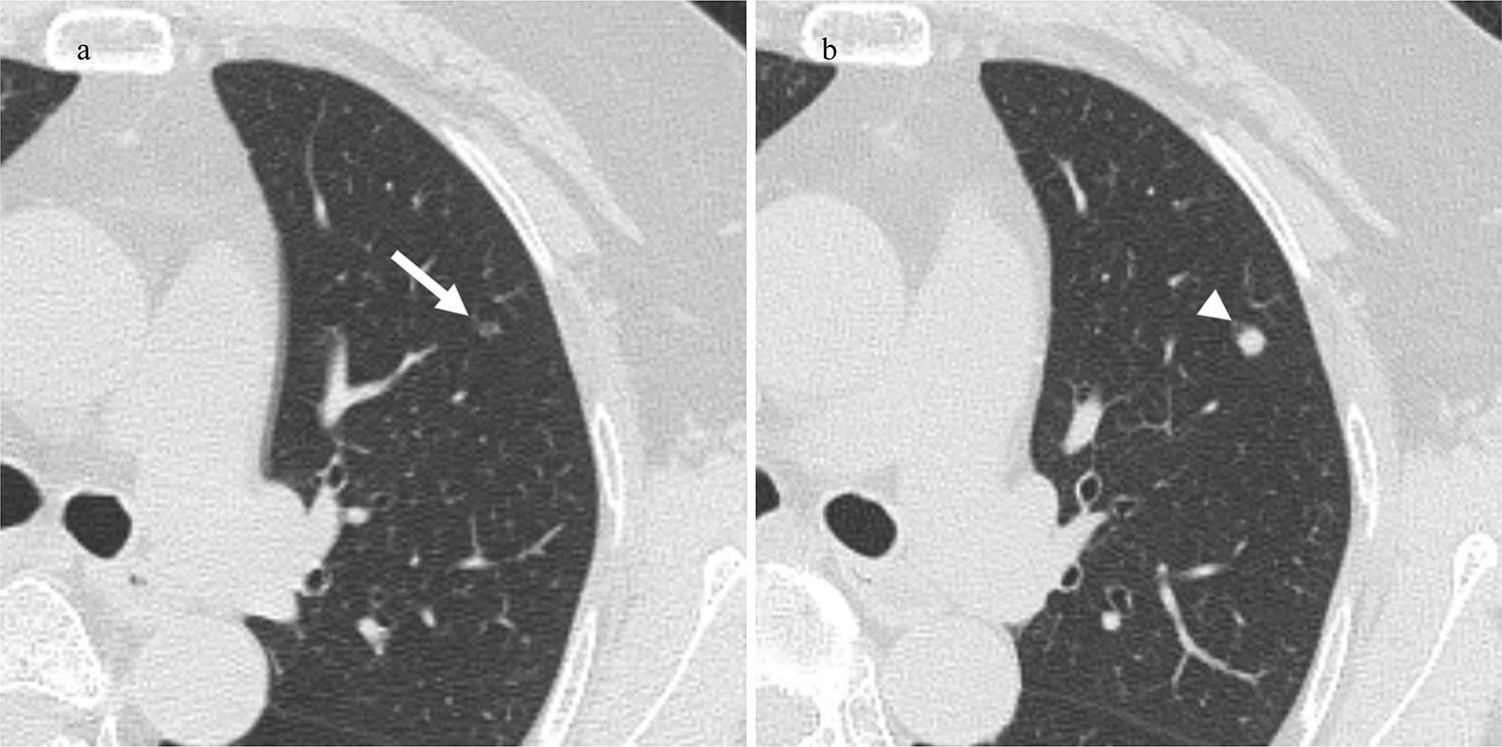
A 72-year-old woman with cutaneous melanoma. **a**. CT after 5 years of operation shows a 3 mm-sized GGN in the left upper lobe (white arrow). **b**. CT obtained 15 months after Fig. 4a shows a GGN that has grown to 5 mm and turned to a solid nodule (white arrowhead). Surgery was performed, and the nodule was diagnosed as a metastasis from malignant melanoma

Of the 13 patients in the GGN group (types 2, 3, and 4), 11 had GGN with an increased size regardless of systemic therapy. Of the 11 patients with GGN with an increased size, six (54.5%) had nodules that changed from GGNs to solid nodules. One patient underwent surgery (Fig. 4).

**Fig. 5:**
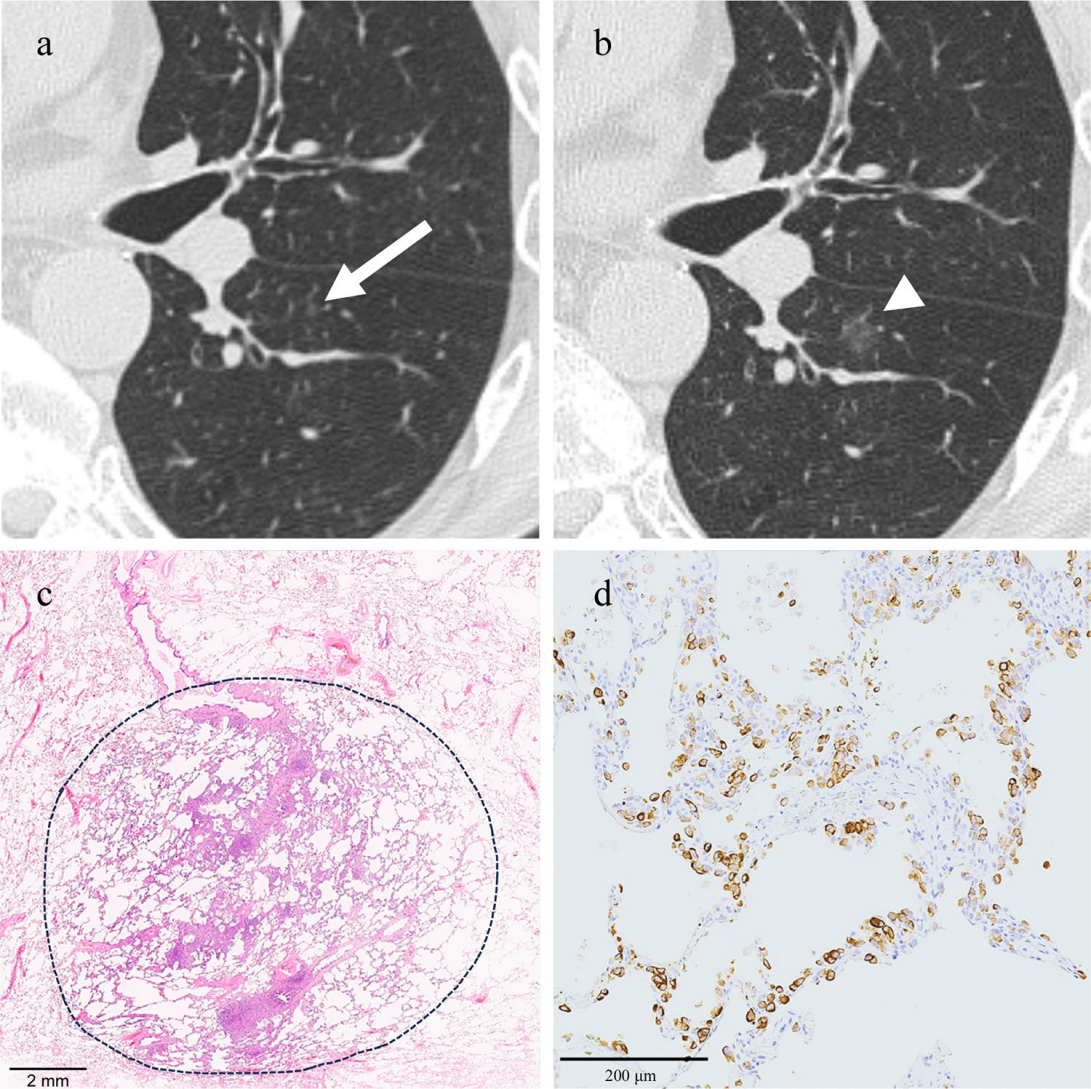
A 65-year-old man with cutaneous melanoma. **a**. CT image shows a 2-mm diameter pure GGN in the left lower lobe (white arrow). **b**. CT image obtained 15 months after Fig. 5a shows that the GGN has grown to 11 mm in diameter (white arrowhead). Surgery was performed. **c**. A low-power histological view of the lung tumor (hematoxylin and eosin staining). Tumor cells spread along the alveolar walls, showing a lepidic growth pattern (dotted line). **d**. HMB45 immunostaining shows melanoma cells replacing the pre-existing alveolar epithelial cells. In this case, three other GGNs grew rapidly, all of which were considered metastases from malignant melanoma (GGN-dominant pattern)

In this study, one more patient underwent surgery. In this case, four fast-growing GGNs were observed. The patient was initially diagnosed with a fast-growing lung adenocarcinoma. One of the four GGNs was surgically resected, and the pathological result revealed pulmonary metastasis of the malignant melanoma; the tumor demonstrated a lepidic growth pattern (Fig. 5).

Compared with the SN group, more patients in the GGN group had mucosal melanomas than acral melanomas (p = 0.0478); however, no significant difference was observed in the frequency between mucosal and cutaneous melanomas (p = 0.0670). Additionally, compared with the SN-dominant pattern, more patients in the GGN-dominant pattern group had mucosal melanomas (p = 0.0342, mucosal melanoma vs. acral melanoma; p = 0.0344, mucosal melanoma vs. cutaneous melanoma; Table 1).

## Discussion

The prognosis of lung metastasis is better than that of other visceral metastases except for skin or distant node metastases. Therefore, lung metastasis is classified separately from other distal metastases in the melanoma staging of the American Joint Committee on Cancer [12, 17]. Several studies showed a poor prognosis of mucosal melanoma compared with that of cutaneous melanoma. Prognosis by clinical subtypes may depend on available agencies [18]. Since *BRAF* V600E/K mutation in mucosal melanoma is less frequent than that in cutaneous melanoma, most of the patients with mucosal melanoma have no treatment options with BRAF/MEK inhibitors. Furthermore, the efficacy of immune checkpoint inhibitors is lower in mucosal melanoma than in cutaneous melanoma [19].

In this study, GGNs were observed in 14.9% of patients with pulmonary metastases from malignant melanoma. We observed five case reports of lung metastases from malignant melanoma that appeared as GGN in previous studies [5–9]. To our knowledge, this is the first study to report on the frequency of lung metastases from malignant melanomas appearing as GGNs. In contrast, lung metastases from pancreatic cancer reportedly have a high frequency of air-space patterns [1–4]. Aissaoui et al. reported that among 76 patients with pulmonary metastasis from pancreatic cancer, three (4%) comprised pure GGN [3]. In this study, four patients (4.6%) had only pure GGN metastasis (Type 4), which is similar to that reported by Aissaoui et al. [3]. At least 10 patients exhibited GGNs in Aissaoui's report (7 with halo signs and 3 with pure GGNs; 10/76 = 13.2%). The frequency of GGN metastasis was 14.9% in our study. Although the classifications used in his study and ours differ, making detailed comparisons challenging, we believe that lung metastases from malignant melanoma may present GGNs approximately as frequently as lung metastases from pancreatic cancer. As previously mentioned, 54.5% of 11 patients with enlarged nodules experienced a change from GGN to solid nodules. Depending on when the follow-up CT was performed, the GGN may have already changed into a solid nodule, which may have affected the frequency of GGNs observed in these studies.

**Fig. 6:**
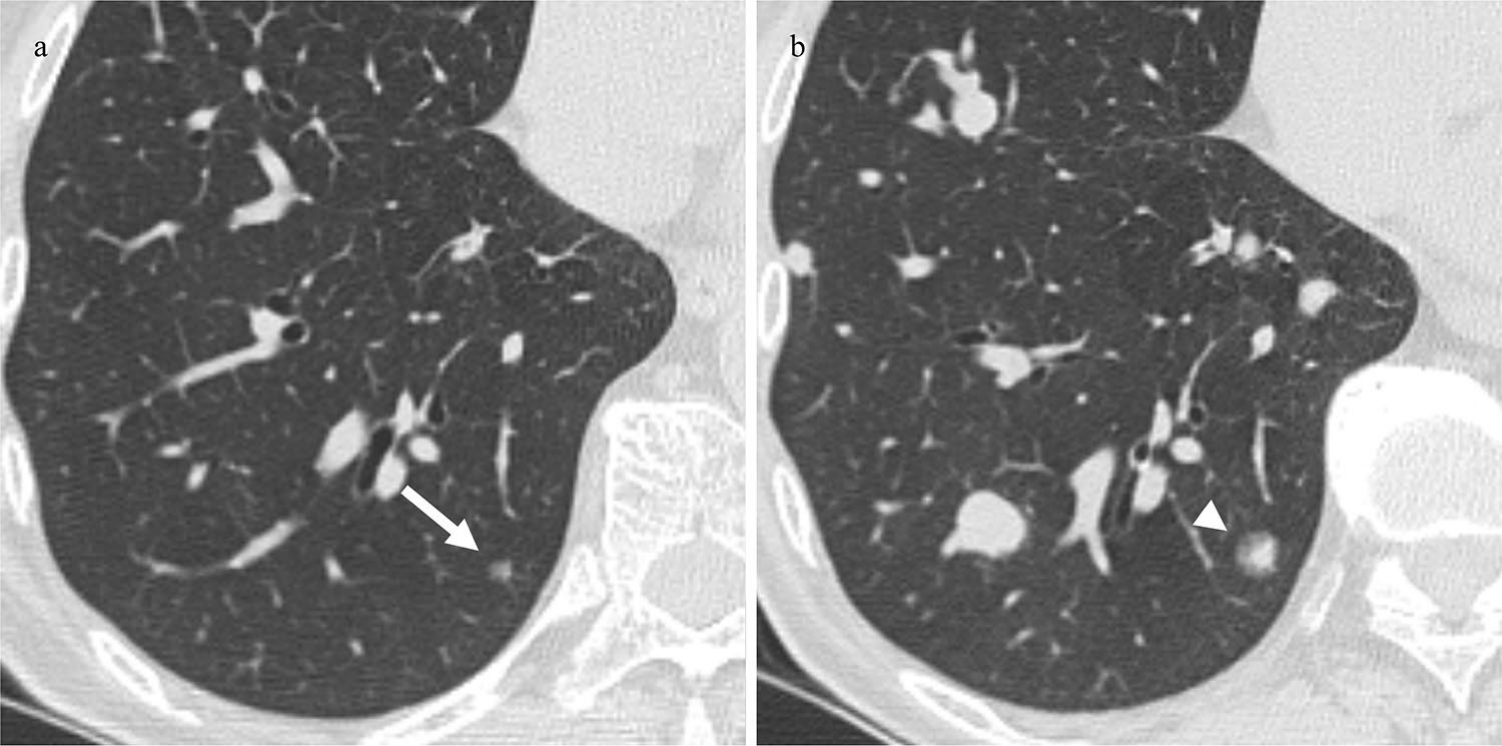
A 67-year-old woman with mucosal melanoma. **a**. CT 24 months after the operation shows 3 mm-sized pure GGN in the right lower lobe (white arrow). **b**. After 6 months, the GGN had grown to 8 mm and turned into a part-solid nodule (white arrowhead). This nodule resembled a lung adenocarcinoma; however, it grew at a faster rate than a lung adenocarcinoma. In addition to this nodule, there were multiple fast-growing GGNs and solid nodules in bilateral lungs (not shown). This nodule was diagnosed as lung metastasis

Various pathologies, including inflammation, focal fibrosis, atypical adenomatous hyperplasia, lung adenocarcinoma, and, rarely, metastatic tumor, present with ground-glass opacity. The most important differential diagnosis for GGN is lung adenocarcinoma. It is difficult to differentiate GGN from lung cancer and metastases from malignant melanoma on CT without follow-up CT (Fig. 6). We believe that the difference in the tumor doubling time is a point of differentiation between metastatic malignant melanoma and lung adenocarcinomas. In our study, the tumor doubling time was 52.0 ± 33.5 days (SD)/50.9 days (median) (range: 10.9–111 days). As for lung metastases of malignant melanoma, Okita et al. reported that multiple GGO lesions increased after 1 month [5]. Mizuuchi et al. reported a solitary GGN with a tumor doubling time of 230 days [6]. Kang et al. reported a solitary GGN that enlarged from 7 to 11 mm in diameter over 3 months [8]. Masuda et al. and Daliaz et al. reported a case of metastatic malignant melanoma identified as a rapidly increasing GGN (the exact tumor size was not described) [7, 9]. In contrast, regarding the rate of tumor growth in lung adenocarcinoma, Aoki et al. reported that Noguchi classifications A, B, and 48% of C had a tumor doubling time of > 1 year [20]. Hasegawa et al. reported mean volume doubling times of 813 and 457 days for pure GGN and part-solid nodules, respectively [21]. Kakinuma et al. reported that 90% of GGNs < 5 mm were stable. They also reported that the duration of the appearance of solid components in adenocarcinomas was 3.6 years [22]. The tumor growth rate of malignant melanoma metastasis is faster than that of lung adenocarcinoma, which may be a differentiating factor.

However, differences in the tumor doubling time should also be reflected in the follow-up period. According to Lung Imaging Reporting and Data System (Lung-RADS) Version 2022, CT examination within 12 months is recommended for non-solid nodules less than 30 mm and part-solid nodules with a total diameter < 6 mm, whereas CT examination within 6 months is recommended for non-solid nodules with a diameter greater than 30 mm, part-solid nodules with a total diameter greater than 6 mm, and solid components with a diameter less than 6 mm [23]. A GGN detected in the lungs of a patient with a history of malignant melanoma indicates a possible metastasis, and using the same follow-up time as in the Lung-RADS Version 2022 may be considerably late. In our study, the tumor doubling time was 52.0 ± 33.5 days; thus, we recommend short-term follow-up at 1–2 months.

Pathologically, hemorrhage and lepidic tumor growth are the most common causes of pulmonary metastases with GGO [1–9, 24, 25]. There are reports of GGN metastases from hemorrhage, including malignant melanoma, angiosarcoma, choriocarcinoma, osteosarcoma, and Kaposi’s sarcoma [1, 24, 25], and those from lepidic tumor growth, including malignant melanoma, pancreatic cancer, colon cancer, breast cancer, and gastric cancer [1–9]. We have only two pathologically proven cases, and there was no hemorrhagic metastasis.

Our findings suggest that lung metastasis with GGN is more likely to occur in mucosal melanomas than in cutaneous or acral melanomas (Table 1). Melanoma types in previous reports were variable and included one mucosal, two cutaneous, one acral, and one uveal melanoma [5–9]. More cases are warranted to determine whether GGNs are more likely to be present in mucosal melanoma.

This study had certain limitations. First, CT examinations were performed using various protocols owing to the retrospective nature of the study. Second, many patients in our study did not have pathologically confirmed pulmonary metastases from malignant melanoma. Third, the number of patients with GGN metastasis was small.

In conclusion, 14.9% of patients with lung metastases of malignant melanoma showed GGNs. Tumor doubling time is useful for differentiating between lung metastasis of malignant melanoma and lung adenocarcinoma. We suggest that short-term follow-up CT should be used to differentiate lung metastases from malignant melanoma and other malignancies that appear as GGNs.
